# Spatiotemporal variation in the interactions between rural tourism and rural settlements in Shanxi and Shaanxi Provinces, China

**DOI:** 10.1371/journal.pone.0318625

**Published:** 2025-04-16

**Authors:** Xiaoqin Geng, Ruiqin Ma, Li Liu, Yixiong Wu

**Affiliations:** 1 College of Public Administration, Shanxi Agricultural University, Jinzhong, China; 2 College of History and Tourism Culture, Shanxi Normal University, Taiyuan, China; 3 College of Economics and Management, Shanxi Normal University, Taiyuan, China,; 4 College of Business,Huaihua University, Huaihua, China; Yogi Vemana University, INDIA

## Abstract

Rural revitalization requires the coordinated development of rural tourism (RT) and rural environments for human settlements. The Chinese provinces of Shanxi and Shaanxi face significant interregional differences as they seek to improve their rural human settlements. This study constructed an index system to evaluate the relationship between RT and rural settlements using the entropy method. The level of coordination between RT and the development of rural settlements in the two provinces during the period 2013–2022 was then analyzed based on the extent of their interaction (i.e., their coupling coordination degree). Key drivers of this coordination were identified using the geographic detector method. The results indicated that the coordination between RT and rural settlements has generally improved in both provinces, but regional disparities remain significant. Central cities are the most developed, while resource-dependent cities and remote areas lag. Critical drivers of coordinated development included economic development, resource attractiveness, and service support. Policy recommendations to promote the balanced development of RT and rural settlements include strengthening the protection of the environment, improving infrastructure, fostering industrial integration, and cultivating human resources.

## 1 Introduction

Rural tourism (RT) is a vital driver of rural revitalization. It has thus played an increasingly significant role in promoting economic development, employment, and environmental protection in rural areas. The Chinese government’s rural revitalization strategy has prioritized RT as a key avenue for rural economic development, as well as a way to increase farmers’ incomes while preserving cultural heritage and ecological balance. The importance of RT development was first highlighted in the 2013 No. 1 Central Document, which advocated for optimizing the use of natural and cultural resources in rural areas through diversified RT measures. The National Rural Revitalization Strategic Plan (2018–2022) also emphasized RT as an integral component of the multi-industry synergy necessary for rural development, with a particular focus on strengthening human settlements, improving rural infrastructure, and enhancing public services. These policy initiatives have significantly boosted the coordination in the development of RT and rural settlements, which has improved living standards and contributed to realizing the country’s goals for rural revitalization.

How RT interacts with rural settlements remains underexplored, however; although existing research has highlighted the dynamic and complex relationships between these two systems [1–2], most studies have focused on the national [3] or provincial levels [4], with limited attention to inter-provincial comparisons. A quantitative analysis of spatiotemporal heterogeneity in the interactions between RT and rural settlements in Shanxi and Shaanxi provinces could thus provide critical insights for sustainable RT development and related policymaking.

## 2 Literature review

The rise and development of RT have provided new pathways and impetus for improving and optimizing rural settlements. Research on this topic has attracted the attention of numerous scholars, but current studies have tended to focus primarily on individual aspects of RT and rural settlements, as well as the correlation between them.

### 2.1 Progress in rural tourism research

RT is a crucial driver for rural revitalization [5]. Most theoretical research on the topic has focused on providing a conceptual definition of RT, but despite multi-dimensional and multi-layered analyses based on different disciplinary backgrounds and research perspectives, no unified definition has yet been developed. This is partially a reflection of the complexity and diversity of RT phenomena, as well as the diverse pathways for its development in different regions and cultures with different historical backgrounds.

Internationally, authoritative institutions such as the European Union and the Organization for Economic Co-operation and Development have defined RT as tourism activities that occur in rural areas, thus emphasizing that rurality is its core distinctive characteristic [6]. This definition highlights the regional characteristics and cultural differences of RT and provides an important reference for the international standardization and normalization of the concept. In China, RT is an important force for promoting rural economic transformation and revitalization [7], and research on the topic has covered multiple fields, including economic development pathways [8], mechanisms for integrating RT and rural industry [9], the synergistic effects of regional management and green development [10], strategies for sustainable RT development [11], and measures for optimized development of the industry [12]. Such studies provide important guidance for constructing theories and approaching the practical application of RT.

### 2.2 Progress in rural settlements research

Rural settlements are the foundation for human survival and development. Research on the topic has primarily been based on the Human Settlements Theory proposed by Doxiadis in 1968, which focuses on changes and laws in the location, landscape pattern, and land use of rural settlements [13–15]. The spatial differences and evolutionary mechanisms of rural settlements have been examined in the context of counter-urbanization [16] alongside the role of planning and remediation in sustainable development [17–18]. The content and models of remediation for rural settlements have recently been examined in depth, and scholars have proposed that these aspects cover multiple dimensions, including the natural environment, living conditions, and infrastructure [19], because remediation involves not only material-level improvements but also non-material aspects such as culture and society [20]. Examinations of typical regions have clarified that the core goal of remediation is to improve the quality of life among residents of rural settlements [21], but there are lingering challenges to improving such settlements, including financial constraints [22], underutilization of technology [23], and limited community engagement [24].

### 2.3 Interaction between rural tourism and rural settlements

RT and rural settlements are related through significant interactions, and their collaborative development and mechanisms for interaction have been considered in existing research. Scholars have constructed evaluation frameworks using models to assess the degree of interaction between systems (i.e., the coupling coordination degree, CCD) to diagnose the dynamic changes in and obstacles to their coordinated interaction [1–4]. CCD models reflect how different elements within a system interact and the level of collaboration among those elements [25]; they have been widely applied to assess systems of different scales and in different regions, covering fields such as the environment [26–29], economy [30–31], social development [32–34], urbanization [35–38], and agriculture [39]. These models have also been used to identify key factors influencing the development of RT and rural settlements [1–4].

The rural economy can be enhanced by optimizing the functionality of the layout for land use and developing RT models such as farm stays and specialty inns [40–43]. The application of Geographic Information System (GIS) technology can provide a scientific basis for the optimal allocation of RT resources [44–46]; however, there are significant differences in the extent to which the interactions of RT and rural settlements are aligned across different regions, and these differences require the customized development strategies tailored to local characteristics [1–4].

**Fig 1:**
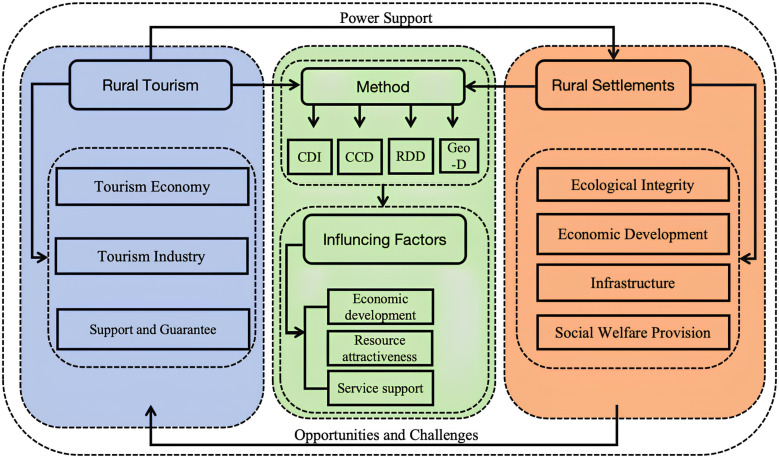
Dynamic mechanism of coordinated interconnections between the development of rural tourism and rural settlements.

Although the integration pathways of RT and rural settlements have been explored, further research is needed to examine the internal mechanisms, cross-regional comparative analyses, and complex interrelationships because this gap affects how policies are formulated and implemented. In response to this issue, this study analyzed the CCD relationship between RT and rural settlements in Shanxi and Shaanxi provinces (Fig 1) by comparing the spatiotemporal differences and discussing the correlation mechanisms in depth to propose optimization strategies for promoting coordinated development.

## 3 Study area and methods

### 3.1 Study area and data sources

**Fig 2:**
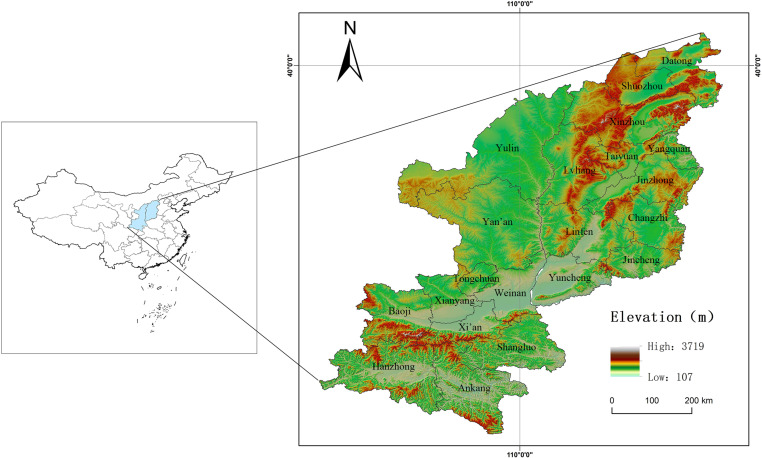
Map of the study area. Reprinted from [47] under a CC BY license, with permission from [SMSS], original copyright [2024]. Note: The map is based on the standard map with review number GS (2024) 0650 downloaded from the Standard Map Service website of the Ministry of Natural Resources [47]; no modifications were made to the base map.

This study focused on Shanxi (34.6°N–40.4°N, 110°E–114.3°E) and Shaanxi (31.7°N–39.6°N, 105.5°E–111.2°E) provinces (Fig 2), which are located in the central Yellow River Basin. Shanxi covers an area of 156,700 km^2^, with 11 prefecture-level cities; in 2022, it had a population of 34.81 million. Shaanxi spans 205,800 km^2^, with 11 cities (excluding the Yangling Demonstration Zone due to data limitations); in 2022, it had a population of 39.56 million. As of 2022, Shanxi Province has more than 30 national-level key RT villages and approximately 200 provincial-level demonstration sites, while Shaanxi Province has 6 national-level key towns, 46 key villages, and 160 provincial-level towns with distinctive features that deeply integrate culture and tourism. Both provinces face challenges related to RT expansion and the sustainability of rural settlements, including water scarcity, ecological fragility, and waste management [23].

The data for this study were taken from the Shanxi Statistical Yearbook, Shaanxi Statistical Yearbook, China Rural Statistical Yearbook, China Cultural Heritage and Tourism Statistical Yearbook, and the China Urban and Rural Construction Statistical Yearbook, as well as official data published by the Ministry of Culture and Tourism, Ministry of Housing and Urban-Rural Development, and the National Forestry and Grassland Administration from 2013 to 2022. Linear interpolation was employed to supply missing data. The data sources can be found in the Data Availability section and S1 File.

### 3.2 Methods

#### 3.2.1 Constructing the evaluation index system.

Relevant research was surveyed across the Web of Science and China National Knowledge Infrastructure (CNKI)databases to ensure the selection of indicators was systematic, comprehensive, and scientifically guided. Indicators with higher usage frequencies (following the frequency count method) were pinpointed, and initial indicators were selected that aligned with the regional characteristics of Shanxi and Shaanxi, as well as indicator availability. The indicators were refined through consultations with domain experts, iterative dialogues, and meticulous deliberations, which allowed us to establish a multidimensional indicator system for evaluating the interrelationships between RT and rural settlements in Shanxi and Shaanxi (Table 1).

**Table 1:** Evaluation index system for rural tourism and rural settlements.

System	Subsystem	Variables & Direction	Weight (Shanxi)	Weight (Shaanxi)	References
RT (U_1_)	Tourism Economic Impact	RT Visits (10,000 people) x1 (+)	0.058	0.076	[4,34]
		Total Income from RT (100,000,000 CYN) x2 (+)	0.063	0.108	[4,34]
		Per Capita Income from RT (Yuan) x3 (+)	0.017	0.025	[4,34]
	Tourism Industry	Number of National-Level RT Sites (Sites) x4 (+)	0.139	0.131	[4,34]
		Number of One Village, One Product Demonstration Villages x5 (+)	0.054	0.046	[34]
		Number of Scenic Spots Rated 3A and Above x6 (+)	0.050	0.047	[1]
		Total Number of Rural Catering and Accommodation Establishments x7 (+)	0.194	0.084	[4]
	Support and Guarantee	Number of Rural Cultural Stations x8 (+)	0.048	0.033	[1]
		Total Collection of Books in Libraries (1,000 items) x9 (+)	0.139	0.156	[1]
		Number of Tourism Schools x10 (+)	0.080	0.159	[4]
		Number of Personnel Engaged in RT x11 (+)	0.158	0.137	[1,34]
Rural Settlements (U_2_)	Ecological Integrity	Household Waste Treatment Coverage (%) y1 (+/−)	0.047	0.049	[4,21]
		Domestic Wastewater Treatment Coverage (%) y2 (+/−)	0.046	0.066	[4,21]
		Greening Rate (%) y3 (+)	0.056	0.070	[1,4]
		Afforestation Area (ha) y4 (+/−)	0.257	0.104	[1]
		Village Rectification Coverage (%) y5 (+)	0.042	0.049	[21]
	Economic Development	Farmers’ Per Capita Net Income (yuan) y6 (+)	0.055	0.068	[1]
		Agricultural GDP (billion yuan) y7 (+)	0.110	0.093	[1,4]
		Education/Culture/Entertainment Expenditure (%) y8 (+)	0.044	0.070	[4]
		Engel’s Coefficient (%) y9 (+)	0.037	0.041	[21]
	Infrastructure	Mobile Internet Broadband Coverage (%) y10 (+)	0.046	0.064	[21,24]
		Toilet Coverage (%) y11 (+)	0.044	0.082	[21,24]
		Per Capita Housing Area (m2) y12 (+)	0.036	0.063	[1,21,24]
	Social Welfare	Medical Staff per Village (people) y13 (+)	0.078	0.072	[4,21,24]
		Safe Drinking Water Coverage (%) y14 (+)	0.045	0.054	[21,24]
		Average Years of Education (years) y15 (+)	0.056	0.056	[21,24]

RT, rural tourism; + and − signify the positive and negative directions of the indicator, respectively, while +/− indicates that the nature of the indicator may exhibit either positive (+) or negative (−) attributes based on the context.

#### 3.2.2 Data standardization.

Given the variations in measurement units, numerical ranges, and positive–negative attributes across indicators, dimensionless processing of the initial data must be conducted to mitigate inherent distinctions. This step ensures that all indicators can be equitably compared and analyzed under a unified standard. The formula for Z-Score normalization (standardization) is as follows:


$$z_{ij}=\frac{X_{ij}-u}{\sigma }$$ (1)

where $x_{ij}$ denotes the initial value of the *j* th indicator in the *i* th year, *u* signifies the arithmetic mean across all observations within the original value dataset, and *σ* denotes the standard deviation—a metric gauging the spread of each data point relative to the mean. $Z_{ij}$ represents the normalized data for the *j* th indicator in the *i* th year.

#### 3.2.3 Comprehensive development index model.

Before quantifying the evaluation system for RT and rural settlements, it is essential to determine the weight of each indicator. Using the entropy method to determine weights helps to reduce any arbitrariness that may be introduced by traditional subjective weighting methods. Higher entropy values signify increased data uncertainty, while lower values indicate more concentrated information and a higher informational contribution [48–49]. The following steps were used in this process. The proportion of the *j* th indicator in the *i* th year was calculated using the following equation:


$$p_{ij}=z_{ij}∕\overset{2022}{\underset{i=2013}{\sum }}z_{ij}$$ (2)

The information entropy of the *j* th indicator was determined using the following equation:


$$E_{\dot{j}}=-{\left(logm\right)}^{-1}\Sigma_{\text{i}=2013}^{2022}p_{ij}$$ (3)

The utility value of the *j* th indicator is given by


$${\text{d}}_{j}=1-E_{j}$$ (4)

The weight of the *j* th indicator is computed as


$$w_{j}={\text{d}}_{j}∕\overset{n}{\underset{j=1}{\sum }}{\text{d}}_{j}$$ (5)

The overall developmental status of the system in the *i* th year can then be represented as


$$S_{i}=\overset{n}{\underset{j=1}{\sum }}w_{i}\times p_{ij}$$ (6)

In these formulas, m denotes the count of evaluation years, while n signifies the number of indicators under consideration. The weight results of each first-class index of the two subsystems from 2013 to 2022 can be found in the Data Availability section and S2 File. Following the data standardization process, the comprehensive development index (CDI) for RT and rural settlements across 21 prefecture-level cities, spanning the period 2013–2022, could be computed using the weighted average methodology.

#### 3.2.4 CCD model.

The CCD model quantitatively evaluates the interdependence and intensity of constraints (i.e., the degree of coupling) among systems, as well as the level of harmonized synergy during this interaction (i.e., the degree of coordination). The following equations were used to develop the CCD model:


$$C=\frac{2\sqrt{U_{1}U_{2}}}{U_{1}+U_{2}}$$ (7)

$$T=\alpha U_{1}+\beta U_{2}$$ (8)

$$D=\sqrt{C\times T}$$ (9)

where *C* represents the coupling degree; *T* represents the comprehensive coordination index between RT and rural settlements; $U_{1}$ and $U_{2}$ represent the comprehensive evaluation indices of RT and rural settlements, respectively; and *α* and *β* are undetermined coefficients, with *α* = *β* = 0.5 due to the equal importance of RT and rural settlements within the system. *D* represents the CCD, where a higher value indicates a more harmonious relationship between RT and rural settlements, and a lower value indicates a noticeable gap between the two systems.

#### 3.2.5 Relative dependency degree model.

While the CCD model can reflect the extent to which the development of two major systems is coordinated, it cannot measure their relative development levels. The relative development degree (RDD) model was therefore introduced:


$$\beta =U_{1}∕U_{2}$$ (10)

The range of *β* and its corresponding practical meaning were determined based on existing research [50]. According to current studies [51], the state of coordinated interaction between the RT and rural settlements systems can be divided into six degrees and three types (Table 2).

**Table 2:** Interactions between rural tourism and rural settlements: types and development stages.

Stage of Development	*D* Value Range for CCD	R	Type
Disorder	[0.0–0.3)	U1 > U2	Rural Settlements Lagging
		U1 < U2	RT Lagging
	[0.3–0.4)	U1 > U2	Rural Settlements Lagging
		U1 < U2	RT Lagging
Integration	[0.4–0.5)	U1 > U2	Rural Settlements Lagging
		U1 < U2	RT Lagging
	[0.5–0.6)	U1 > U2	Rural Settlements Lagging
		U1 < U2	RT Lagging
Coordination	[0.6–0.7)	U1 > U2	Rural Settlements Lagging
		U1 < U2	RT Lagging
	[0.7–1.0]	U1 > U2	Rural Settlements Lagging
		U1 < U2	RT Lagging

CCD, coordinated coupling degree; U1 and U2, the comprehensive evaluation indices of RT and rural settlements, respectively; and RT, rural tourism.

#### 3.2.6 Geographical detector.

The geographical detector can support the analysis of the mechanisms and individual factors that affect RT and rural settlements, as well as the interaction effect of two factors. The geographical detector has no hypothesis conditions, which effectively reduces the limitations of handling causal relationships with general statistical analysis methods [52]. Following prior research [53], it can be expressed as


$$q=1-\frac{1}{N\delta^{2}}\Sigma_{i=1}^{L}N\dot{i}\delta_{i}^{2}$$ (11)

where N is the total number of samples in the study area; ${\text{δ}}^{2}$ is the total variance in the study area; L is the number of sub-regions; and Ni and ${\text{δ}}_{i}^{2}$ are the sample numbers and variances in region i. The value of q ranges from 0 to 1 and reflects the degree of spatial differentiation. A higher q value indicates a stronger influence of the factor on the CCD model.

## 4 Results and analysis

### 4.1 Spatiotemporal variations of CDI in rural tourism and rural settlements

#### 4.1.1 Temporal trends.

**Fig 3:**
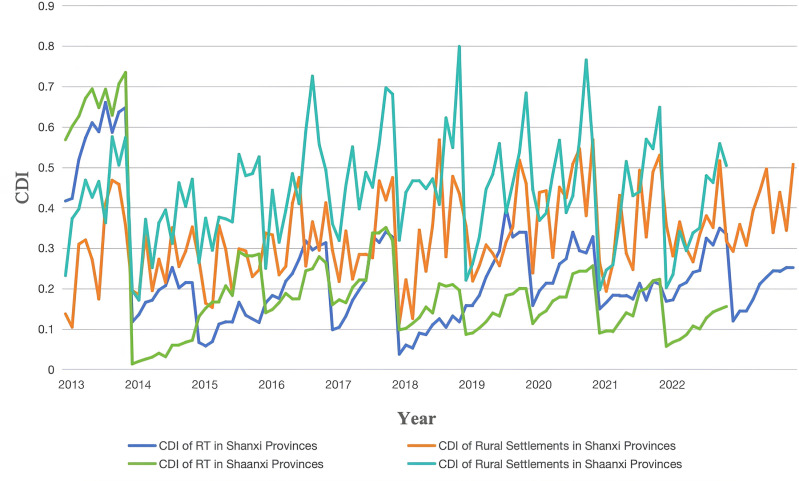
Comprehensive development index values for rural tourism and rural settlements in Shanxi and Shaanxi Provinces, 2013–2022.

By collecting panel data from 2013 to 2022, we calculated the CDI levels of RT and rural settlements (Fig 3). During this period, most regions experienced an upward trend in these indices, but the growth rates varied significantly across different stages. In 2015, the opening year of the “13th Five-Year Plan,” development in most cities accelerated, which led to a significant increase in CDI values. In 2020, due to the impact of the COVID-19 pandemic, most RT CDI values declined to varying degrees, while in 2022, the post-pandemic recovery boosted most indices, but the level of divergence intensified.

1. Regions with Synchronous Growth. Taiyuan and Xi’an saw continuous increases in the RT and rural settlements indices from 2013 to 2022. Xi’an’s rapid RT growth benefited from policy support as a core area of the Belt and Road Initiative and industrial upgrades, while the significant improvement in the rural settlements index values reflected continuous improvements in healthcare, education, and living standards. The growth rates of RT and rural settlements were particularly synchronized from 2016 to 2017, thus demonstrating the economic pull effect on social quality.
2. Regions with Faster RT Growth. Datong and Jincheng saw RT indices rise from 0.117 and 0.097 to 0.2120 and 0.327 over ten years, respectively. Overall, RT indices improved, but rural settlements growth was minimal. This indicates that economic growth did not fully translate into synchronous social development, and this failure may be associated with insufficient social investment in the economic models of resource-based cities.
3. Regions with Fluctuating Rural Settlements. Xianyang’s rural settlements peaked at 0.589 in 2019 but dropped to 0.556 in 2020 and fell further to 0.492 in 2022. This decline was linked to the impact of the COVID-19 pandemic on the distribution of medical, educational, and social resources—especially the interruption of social public services during the 2020 pandemic. Yan’an’s rural settlements index value reached 0.622 in 2020 but rose rapidly to 0.798 in 2022, thus reflecting a significant increase in social resource investment in that year.

#### 4.1.2 Spatial distribution.

**Fig 4:**
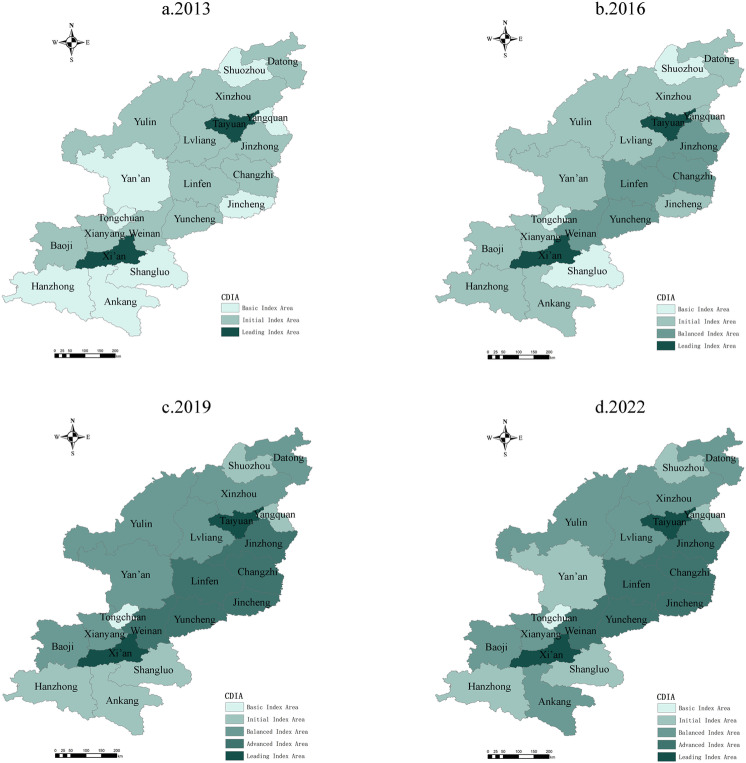
Spatial Pattern for the Distribution of Rural Tourism Comprehensive Development Index Values in Shanxi and Shaanxi Provinces. Reprinted from [47] under a CC BY license, with permission from [SMSS], original copyright [2024]. Note: The map is based on the standard map with review number GS (2024) 0650 downloaded from the Standard Map Service website of the Ministry of Natural Resources [47]; no modifications were made to the base map.

**Fig 5:**
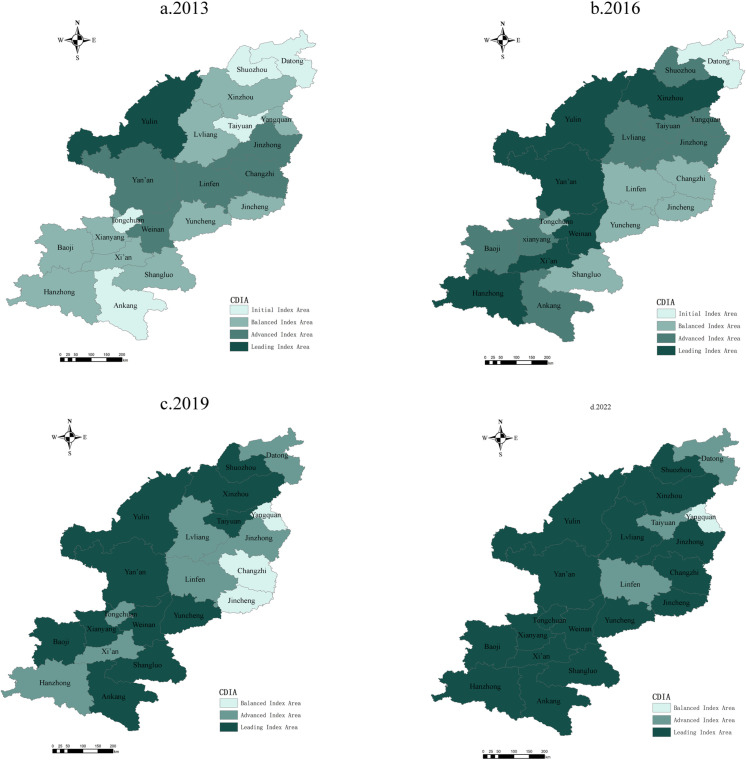
Spatial distribution of rural settlements comprehensive development index values in Shanxi and Shaanxi provinces. Reprinted from [47] under a CC BY license, with permission from [SMSS], original copyright [2024]. Note: The map is based on the standard map with review number GS (2024) 0650 downloaded from the Standard Map Service website of the Ministry of Natural Resources [47]; no modifications were made to the base map.

To observe regional differences, the natural breaks technique divided the CDI values for rural settlements and RT in Shanxi and Shaanxi into five levels: basic, initial, balanced, advanced, and leading (Table 3). ArcGIS 10 software was then used to illustrate the spatial distribution of CDI in the prefecture-level cities in Shanxi and Shaanxi (Figs 4 and 5), which followed a pattern in which the regional central cities were in the lead, while the surrounding cities lagged. Provincial capital cities had significantly higher CDI values, while resource-based cities and remote areas lagged, thus highlighting regional disparities.

**Table 3:** Comprehensive development index levels for rural tourism and rural settlements in Shanxi and Shaanxi provinces.

Level for Area Comprehensive Development Index	Value
Basic	[0, 0.1)
Initial	[0.1,0.2)
Balanced	[0.2,0.3)
Advanced	[0.3,0.4)
Leading	[0.4,1]

1. Regional Center Cities Lead Development. Xi’an and Taiyuan, as provincial capitals, consistently led in CDI. In 2022, Xi’an’s RT index reached 0.734, and Taiyuan’s was 0.647; these scores were far higher than those of other cities in their respective provinces. Both cities showed significant advantages in the rural settlements indices, with Xi’an reaching 0.573 and Taiyuan at 0.356. This advantage appeared to stem from factors including resource concentration and policy support in economics, science and technology, and education, and especially the significant development dividends brought by the Belt and Road Initiative to Xi’an.
2. Lagging Resource-Based Regions. Cities such as Yangquan in Shanxi and Tongchuan in Shaanxi lagged in the RT and rural settlements indices. For example, Datong’s RT index was only 0.212 in 2022, and the rural settlements CDI value was 0.352; these values are much lower than the average levels in both provinces. This lag can be attributed to insufficient social development driven by resource-based economies and inadequate investment in social services during economic transitions.
3. Significant Regional Disparities. Regional disparities were prominent in both provinces. In Shanxi, the cities of Taiyuan, Changzhi, and Jinzhong were relative leaders, while Yangquan and Shuozhou lagged. In Shaanxi, cities in the Guanzhong Plain such as Xi’an, Xianyang, and Baoji developed faster, whereas cities in northern (e.g., Yulin) and southern Shaanxi (e.g., Shangluo and Ankang) lagged, especially in rural settlements values, which reflects the more pronounced regional imbalances in social sectors and, in particular, the differences in infrastructure, economic structure, and resource endowments across regions.

### 4.2 Spatiotemporal variations in CCD values

#### 4.2.1 Temporal trends.

**Fig 6:**
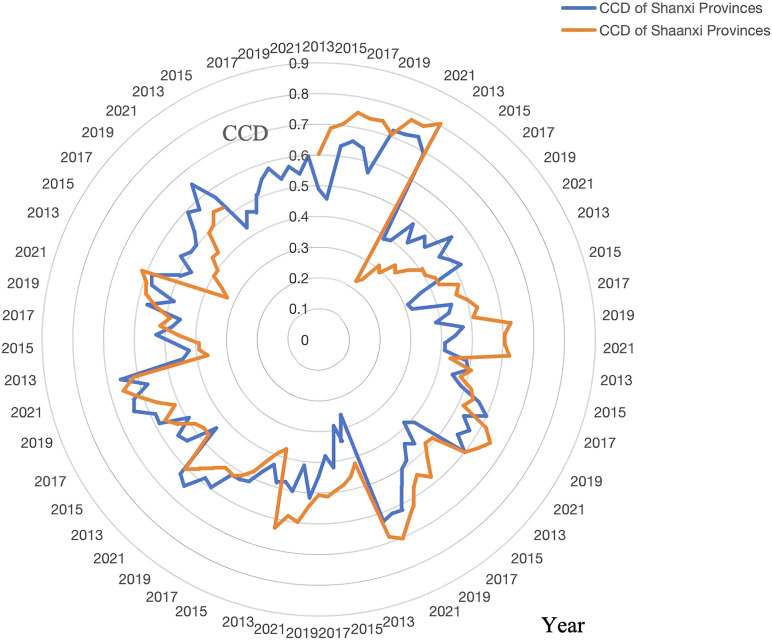
Coupling coordination degree values in Shanxi and Shaanxi Provinces, 2013–2022.

From 2013 to 2022, the CCD between RT and rural settlements in Shanxi and Shaanxi showed a significant improvement (Fig 6). The final calculation results of coupling and coordination model from 2013 to 2022 are shown in S3 File. Shanxi’s CCD steadily progressed from the slight imbalance stage to moderate coordination, while Shaanxi rapidly advanced from the slight imbalance stage to high coordination. However, the performance of the two provinces varied at different periods, influenced by policies and external events. The speed of recovery and the degree to which coordination was enhanced during the pandemic had a particularly pronounced impact.

1. Steady Improvement in Shanxi’s CCD. From 2013 to 2019, policy support and infrastructure investment significantly promoted the coordinated development of RT and rural settlements. In 2019, Taiyuan entered the high coordination stage and became a provincial benchmark. However, after 2020, Shanxi’s overall CCD experienced a setback due to the COVID-19 pandemic, especially in resource-based cities such as Yangquan and Shuozhou, which recovered slowly. The pandemic affected local economies and policy execution, which limited collaborative improvements to RT and rural settlements.
2. More Significant Growth in Shaanxi’s CCD. Shaanxi’s CCD grew more significantly, especially during the post-pandemic recovery phase. In 2014, Shaanxi entered the high-quality coordination stage and continued to improve with subsequent policy support. From 2017 to 2022, cities such as Yan’an and Weinan leveraged eco-tourism development and infrastructure improvements to increase their level of coordination, becoming models of provincial coordinated development. In contrast, although Xi’an and Xianyang had higher starting levels, their rates of growth were relatively slower due to environmental pressures and challenges in industrial upgrading.

#### 4.2.2 Spatial distribution.

**Fig 7:**
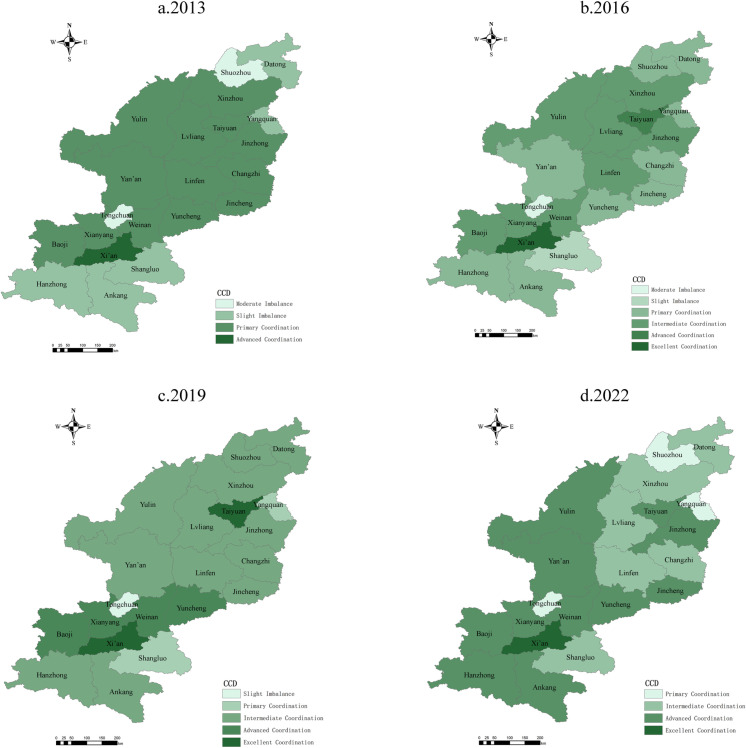
Spatial pattern of coordinated development of rural tourism and settlements in Shanxi and Shaanxi provinces. Reprinted from [47] under a CC BY license, with permission from [SMSS], original copyright [2024]. Note: The map is based on the standard map with review number GS (2024) 0650 downloaded from the Standard Map Service website of the Ministry of Natural Resources [47]; no modifications were made to the base map.

As visualized using ArcGIS10 software (Fig 7), the spatial distribution of CCD values for RT and rural settlements in Shanxi and Shaanxi provinces exhibits significant regional disparities. The final calculation results of coupling and coordination model from 2013 to 2022 are shown in S3 File. These variations reflect the distinct local characteristics in resource endowments, economic structures, and policy implementations within the two provinces. In Shanxi Province, a pattern characterized by central dominance and peripheral faltering is evident, whereas Shaanxi Province seems to be following a model of core leadership with radiating improvements.

1. Shanxi Province. The spatial distribution of CCD values centered around major cities such as Taiyuan and Jinzhong, where stronger economic foundations and infrastructure advantages contributed to higher levels of coordination. Peripheral resource-dependent cities such as Datong and Yangquan exhibited lower CCD values, thus highlighting issues related to the pressures of industrial transformation and inadequate infrastructure. Resource-rich cities such as Lvliang and Changzhi, despite their development potential, also experienced considerable fluctuations in CCD values due to imbalances between rapid growth in RT and improvements to social services.
2. Shaanxi Province. The spatial distribution of CCD values showed higher equilibrium and overall better performance in Shaanxi than in Shanxi. Cities surrounding Xi’an, such as Yan’an and Weinan, have seen continuous improvement in CCD values due to policy support and eco-tourism development. Xi’an is the provincial capital and tourism hub; it maintains high-quality coordination but faces constraints on growth due to environmental pressures from urban expansion and challenges in industrial upgrading. Peripheral cities have become crucial drivers of CCD growth in Shaanxi Province through ecological resource use and brand building.
3. Comparative Analysis. The spatiotemporal pattern in Shanxi reflects its uneven policy execution and infrastructure investment, and its CCD values are further restricted by poor transportation in northern regions, which limits the synergistic development of RT and rural settlements. Conversely, Shaanxi Province has leveraged stronger economic foundations and policy advantages to form a promising development model centered on Xi’an; the province as a whole thus significantly outperformed Shanxi in overall coordination.

### 4.3 Key drivers of rural tourism–rural settlement coordination

**Fig 8:**
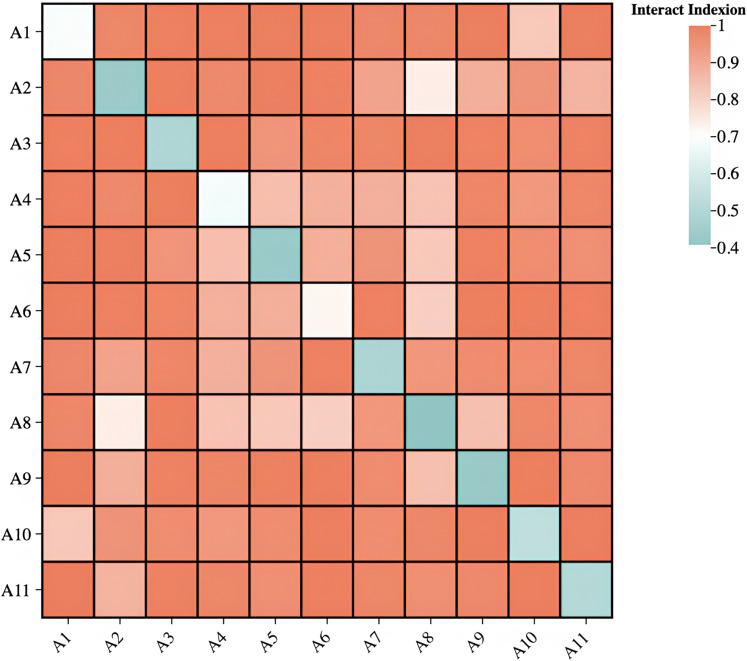
Multi-factor interaction: coordinated development of rural tourism and settlements.

The CCD levels for RT and rural settlements are shaped by multiple factors. This study investigated the causal mechanisms of three primary driving forces using the geographic detector method: economic development, resource attractiveness, and service support. Economic development provides the foundation for optimizing public resources and upgrading services—that is, it provides essential financial support [54]. Key indicators for measuring economic drivers included annual tourism income; per capita net rural income; and total output values for agriculture, forestry, animal husbandry, and fishery. In less developed areas, tourism is a vital emerging industry for promoting regional development [55]. Resource attractiveness was evaluated using indicators such as the number of national-level RT sites; the One Village, One Product demonstration villages; scenic spots above 3A grade; and cultural stations. Access to public facilities and services becomes crucial as rural settlements improve [56], and service support was assessed through indicators such as rural mobile internet coverage, the distribution rate of sanitary toilets, the number of distinctive catering and accommodation outlets, and the accessibility of safe drinking water. SPSS 26.0 was used to discretize continuous variables and perform classification analysis. The single-factor impact and interaction degree of each driving factor on the CCD values of both systems were calculated, thus providing specific data for Shanxi and Shaanxi (Table 4) and visualizations (Fig 8).

**Table 4:** Results of the factor analysis of the coordination in the development of rural tourism and settlements.

Factor	Indicators	q
Economic Development	Total Income from RT (100,000,000 CYN)	0.682
	Farmers’ Per Capita Net Income (yuan)	0.440
	Agricultural GDP (billion yuan)	0.492
Resource Attractiveness	Number of National-Level RT Sites (Sites)	0.663
	Number of One Village, One Product Demonstration Villages	0.434
	Number of Scenic Spots Rated 3A and Above	0.729
	Number of Cultural Stations	0.490
Service Support	Mobile Internet Broadband Coverage (%)	0.408
	Toilet Coverage (%)	0.434
	Total Number of Catering and Accommodation Establishments	0.538
	Safe Drinking Water Coverage	0.510

RT, rural tourism.

1. Economic factors are the drivers of changes in CCD values. The indicator per capita expenditure on RT achieved a q-value of 0.682, which indicates that tourism consumption capacity directly reflects the economic vitality and market appeal of rural areas, thus positively affecting the coordinated development of RT and rural settlements. The q-values for per capita net income of farmers and total output value of agriculture, forestry, animal husbandry, and fishery were 0.439 and 0.492, respectively, thus highlighting the importance of farmers’ income and the agricultural economy. However, their effects on non-agricultural industries such as tourism and culture are limited, thus calling for enhanced industrial integration to boost the contribution of agriculture to rural development.
2. Resource attractiveness provides the foundation for CCD. The highest q-value (0.729) for the number of scenic spots above 3A grade reflects the differences in rural resource endowment and development levels; it thus plays a crucial role in enhancing the coordination of RT and rural settlements development. The q-values for national-level RT sites (0.662) and rural cultural stations (0.489) were also high, which indicates the significant impacts of tourism site creation and construction on the economic, cultural, and ecological development of surrounding rural areas. However, the q-value for demonstration villages in the One Village, One Product program was 0.434, which suggests that the effect of cultural resource development in promoting coordinated development has not yet been fully realized, thus requiring deeper exploration and integrated development of cultural resources.
3. Service support guides CCD. The highest q-value (0.537) for the number of distinctive rural catering and accommodation outlets indicates that service facilities are crucial for the coordinated development of RT and rural settlements, thus reflecting the reception capability and service quality for tourists. The q-values for safe drinking water (0.509) and sanitary toilets (0.434) highlight the significant impact of infrastructure on rural development. Basic public services play a vital role in improving rural living environments and attracting people. The q-value for rural mobile internet broadband coverage was 0.408, which is relatively low but with substantial potential contributions as rural digital access improves, thus gradually enhancing the promotion of rural economic development through informatization.

From the results of the **interaction detection** (Fig 8), all influencing factors exhibited enhancing or nonlinear enhancing interactions, thus highlighting their strong interplay in collectively driving rural development. The interaction between resources and services was most prominent, with an interaction q-value of 0.956 for national RT sites and the number of catering and accommodation establishments. This demonstrates the significant impact of combining resource attractiveness with service compatibility while emphasizing that a high-quality service system greatly enhances the efficiency of resource use. The second most notable interaction was between economy and service, with a q-value of 0.951 for farmers’ per capita net income and the number of catering and accommodation establishments. This reflects a positive feedback loop between improving farmers’ income and the growth of the service industry, which significantly promotes the development of rural settlements. The interaction between the economy and resources was also notable, with a q-value of 0.811 for per capita tourism expenditure and the number of 3A and above tourist attractions. This confirms the strong driving effect of combining the capacity for economic consumption with high-quality tourism resources, thus underscoring the essential role of RT resources in attracting consumption and boosting the rural economy.

## 5 Conclusions

This study constructed a comprehensive evaluation index system for RT and rural settlements based on the entropy method and analyzed the level of coordinated development between RT and rural settlements in Shanxi and Shaanxi provinces from 2013 to 2022 using the CCD model. The key driving factors affecting the coordinated development of the two systems were explored with the geographic detector. The results allowed us to draw three main conclusions.

1. First, the CDI values for RT and rural settlements in Shanxi and Shaanxi provinces had significant spatiotemporal differences. Regional central cities (such as Xi’an and Taiyuan) were development leaders, while resource-based cities and remote areas lagged significantly, thus highlighting pronounced regional disparities.
2. Second, the coordinated development of RT and rural settlements in both provinces generally showed an upward trend. Shaanxi Province performed notably better, especially in its quick recovery from the COVID-19 pandemic. Shanxi Province also improved, but it faced a slower recovery in some cities due to pandemic shocks and pressures from resource-based economic transitions.
3. Third, economic development, resource attractiveness, and service support were the key factors influencing the coordination of development between RT and rural settlements. The following factors significantly propelled rural development: the combination of economic consumption capacity with high-quality tourism resources, the synergy between resource attractiveness and service compatibility, and the positive interaction between farmers’ income and service sector development.

## 6 Discussion and policy implications

### 6.1 Discussion

The coordinated development of RT and rural settlements currently occupies a core position in China’s rural revitalization strategy. Shanxi and Shaanxi are provinces rich in tourism resources and thus face numerous challenges in advancing RT development. The spatiotemporal analysis revealed the characteristics of imbalanced RT development and disparities in improvements to rural settlements. The CCD model was used to analyze the coupling relationship and spatiotemporal heterogeneity between the two systems in depth, thus providing a scientific basis for more precise policymaking. This could not only help promote balanced RT development and comprehensive improvement to rural settlements but could also offer a valuable reference for implementing China’s rural revitalization strategy.

Consistent with existing research [1–4], the development of RT and rural settlements in Shanxi and Shaanxi provinces has generally shown an upward trend. This study particularly highlights that determining how to transcend geographical boundaries to achieve deep integration between RT and rural settlements remains a serious challenge. It is important to face this challenge because such integration would comprehensively promote the diversification and sustainable development of rural industries. During the study period, although the CCD levels between RT and rural settlements improved, an unbalanced development trend overall was still apparent. The possible reasons for this lie in regional differences in policy orientation and enforcement intensity. Since 2017, Shanxi Province has implemented the Rural Revitalization Strategic Plan, which has focused on advancing infrastructure construction and the development of characteristic agriculture. Meanwhile, Shaanxi Province has vigorously promoted ecological tourism and cultural brands through its Province-Wide Tourism Demonstration Construction policy, thus enhancing the development and use of RT resources. The differences in regional policy orientations have, however, led to uneven development; for example, resource-based cities have underinvested in RT and rural settlements during industrial transformation, which has affected coordinated development.

The integration between RT and rural settlements has not yet been achieved in various regions. This has resulted in insufficient industrial diversification and a weak foundation for sustainable development. Future work should focus on strengthening the degree of coordination between RT and rural settlements by implementing precise policy guidance and close collaboration between regions. This approach would effectively integrate resources to promote the diversified development of RT and solidify the foundation for the sustainable development of rural settlements.

### 6.2 Policy recommendations

This study provided beneficial explorations for sustainable rural development, thus contributing to the coordinated development of the economy, society, and environment in rural areas. In doing so, it can offer theoretical support and practical guidance for achieving rural revitalization goals. Based on our results, we propose the following four key policy recommendations to promote the dynamic balance between RT and rural settlements, achieve efficient resource sharing, and drive the coordinated development of the two systems:

1. Economic Integration and Industrial Upgrading. The prosperity of RT depends on integrating agriculture with the tourism and cultural industries. RT enterprises should be encouraged to adopt technological innovations and upgrade products and services to enhance market competitiveness. Human resources should be cultivated in rural areas through skills training and entrepreneurship guidance to improve farmers’ professional and entrepreneurial abilities. Farmers’ income should be increased via policies and funding, thus boosting RT market vitality and ensuring the sustainable, healthy development of the RT industry.
2. Cultural Resource Exploration and Enrichment of Tourism Content. Protecting and exploring rural cultural resources is vital for increasing the appeal of RT. Cultural festivals and folk activities should be organized to showcase rural cultural charm and enrich tourist experiences. Place-specific cultural tourism products should be developed, such as rural cultural experiences and folk custom tours. This would meet tourists’ cultural demands, diversify RT, and enhance the industry’s cultural depth and attractiveness.
3. Improvement of Infrastructure and Service Quality. Upgrading the RT service infrastructure is crucial for improving the tourist experience. Investment in rural infrastructure should be increased, with a focus on transportation, utilities, and communications to enhance accessibility and comfort. The training and management of RT service providers should be strengthened to improve service quality and professionalism. Rural sanitation and living conditions should be enhanced to provide tourists with cleaner, more comfortable travel experiences, thus boosting RT’s overall competitiveness.
4. Cross-Provincial Cooperation and Resource Sharing Mechanisms. Cooperation across administrative boundaries should be strengthened to promote cross-regional RT development. Agreements on planning, product development, and marketing should be signed, while joint meetings should be held to address challenges. Collaboration should be encouraged to improve service facilities, optimize routes, and enhance tourism transportation comfort and convenience, thus achieving resource sharing and mutual guest delivery.

Despite the in-depth analysis of the coordinated development of RT and rural settlements in Shanxi and Shaanxi provinces, this study has some limitations. Notably, it is primarily focused on higher levels and lacks micro-level consideration of towns and villages, which play a critical role in the development of RT and rural settlements. Future research should delve into the town and village levels by acquiring firsthand data through field surveys and questionnaires to further evaluate the effectiveness of the implementation of rural revitalization policies.

This article does not involve any ethical issues, because we have not studied any human or animal subjects, nor have we collected any personal information or sensitive data.

## Supporting information

S1 File Origin data of two subsystems for 21 cities of Shanxi and Shaanxi provinces in China from 2013 to 2022. (XLSX)

S2 File All weight results calculated by entropy method. (XLSX)

S3 File The final calculation results of coupling and coordination model. (XLSX)
